# Development and psychometric testing of Holistic Clinical Assessment Tool (HCAT) for undergraduate nursing students

**DOI:** 10.1186/s12909-016-0768-0

**Published:** 2016-09-22

**Authors:** Xi Vivien Wu, Karin Enskär, Lay Hoon Pua, Doreen Gek Noi Heng, Wenru Wang

**Affiliations:** 1 Alice Lee Centre for Nursing Studies, Yong Loo Lin School of Medicine, National University of Singapore, Level 2, Clinical Research Centre, Block MD 11, 10 Medical Drive, Singapore, 117597 Singapore; 2 School of Health and Welfare, Jönköping University, Jönköping, Sweden; 3 Department of Education and Practice, Nursing Service, Tan Tock Seng Hospital, Singapore, Singapore; 4 Nursing Education, National University Hospital, Singapore, Singapore

**Keywords:** Holistic clinical assessment tool, Tool development, Psychometric testing, Undergraduate nursing students

## Abstract

**Background:**

A major focus in nursing education is on the judgement of clinical performance, and it is a complex process due to the diverse nature of nursing practice. A holistic approach in assessment of competency is advocated. Difficulties in the development of valid and reliable assessment measures in nursing competency have resulted in the development of assessment instruments with an increase in face and content validity, but few studies have tested these instruments psychometrically. It is essential to develop a holistic assessment tool to meet the needs of the clinical education. The study aims to develop a Holistic Clinical Assessment Tool (HCAT) and test its psychometric properties.

**Methods:**

The HCAT was developed based on the systematic literature review and the findings of qualitative studies. An expert panel was invited to evaluate the content validity of the tool. A total of 130 final-year nursing undergraduate students were recruited to evaluate the psychometric properties (i.e. factor structure, internal consistency and test-retest reliability) of the tool.

**Results:**

The HCAT has good content validity with content validity index of .979. The exploratory factor analysis reveals a four-factor structure of the tool. The internal consistency and test-retest reliability of the HCAT are satisfactory with Cronbach alpha ranging from .789 to .965 and Intraclass Correlation Coefficient ranging from .881 to .979 for the four subscales and total scale.

**Conclusions:**

HCAT has the potential to be used as a valid measure to evaluate clinical competence in nursing students, and provide specific and ongoing feedback to enhance the holistic clinical learning experience. In addition, HCAT functions as a tool for self-reflection, peer-assessment and guides preceptors in clinical teaching and assessment.

**Electronic supplementary material:**

The online version of this article (doi:10.1186/s12909-016-0768-0) contains supplementary material, which is available to authorized users.

## Background

A major focus in nursing education is on the judgement of clinical performance, and it is a complex process due to the diverse nature of nursing practice [1]. Difficulties in the development of valid and reliable assessment measures in nursing competency have resulted in the development of assessment instruments with an increase in face and content validity, but few studies have tested these instruments psychometrically [2]. Literature reviews suggest that there is no ‘gold standard’ for measuring clinical competence; thus, assessing nurses’ competency continues to pose a challenge in nursing education [3].

While the assessment of clinical competence requires explicitly defined standards meeting national standards of the nursing profession [4], the standards should be easily understood and interpreted in the same manner by the preceptors, provide a guideline for the nursing students, and be practically applied in the clinical setting. Gonczi [5] advocated a holistic approach in the assessment of competency which combines the knowledge, skills and attitudes of professionals in clinical situations, and this notion of competence incorporates professional judgement, which involves complex structuring, bringing together disparate attributes and tasks required for intelligent performance in a particular clinical situation. Therefore, it is essential to develop a holistic assessment tool to meet the needs of clinical education.

Transition to Practice (TTP) is a consolidated clinical practicum which consists of 9-week clinical practice and assessment for pre-registered nursing students in the university in Singapore. It prepares students to develop the required level of competency to function as beginning practitioners upon licensure registration [6]. According to the American Association of Colleges of Nursing [7], clinical practicum provides opportunities for nursing students to learn in multiple care settings and receive appropriate guidance that fosters the development of clinical competence and professionalism. Nursing students are equipped with theoretical knowledge and beginners’ skill competency throughout the undergraduate study. However, they may lack confidence and clinical experiences [4]. In fact, research demonstrates that new graduate nurses’ confidence depends on the time spent during undergraduate clinical placements and the provision of workload of patients in conjunction with a preceptor to consistently improve issues of time management, competence and confidence with nursing tasks [8].

Clinical competence is defined as the theoretical and clinical knowledge, incorporating psychomotor skills and problem-solving ability with the goal of safely providing care to patients in nursing practice [9]. Nursing is recognised as a reputable profession internationally. Professional bodies are set up to provide guidelines for nursing practice and education. The Nursing and Midwifery Board of Australia [10] defines competency standards for a registered nurse (RN) as the combination of skills, knowledge, attitudes, values and abilities that underpin effective and/or superior performance in a profession. The Singapore Nursing Board [11] emphasises that core competencies set the foundation for RNs to maintain their competence and acquire additional competencies or advanced clinical skills to deliver safe client care in response to changing health-care needs and advancement in technology.

The preceptor model is used commonly in nursing education. This model allows the student to experience the realities of the nurse’s role while practising the skills [9]. Preceptors are registered nurses who work in patient care and student supervision simultaneously, and an educational course is usually conducted by individual healthcare institutions to ensure adequate preparation for the preceptors [12]. The preceptor’s role is to facilitate the learning, build a supportive clinical learning environment, assess the clinical competency of the nursing students, review the progress and provide effective feedback to the students. Preceptors need to possess a strong familiarity with the principles of teaching and learning to effectively facilitate students’ clinical learning [13]. One of the common challenges faced by preceptors is unfamiliarity with the theoretical knowledge and skills taught in the academic institution and the assessment system. This may influence the preceptors’ ability to help students to bridge the gap between theory and practice.

The assessment system is developed by nursing academics, and preceptors are the assessors. Ideally, there should be strong collaboration between academics in the teaching institution and preceptors in the clinical setting. Achieving clinical competency in nursing education is a key element in the development of professional standards and patient safety [14]. Assessment of clinical competence is a crucial task for nursing educators and administrators. The current trend of moving from a generic to a holistic model of assessment of clinical competence supports the development of competent nursing professionals [2]. However, lecturers, preceptors and nursing students often have different interpretations of the assessment system [15]. Hence, it is necessary to develop a valid and reliable holistic clinical assessment tool [16]. This tool could measure the learning gains and enrich the learning process.

**Fig. 1:**
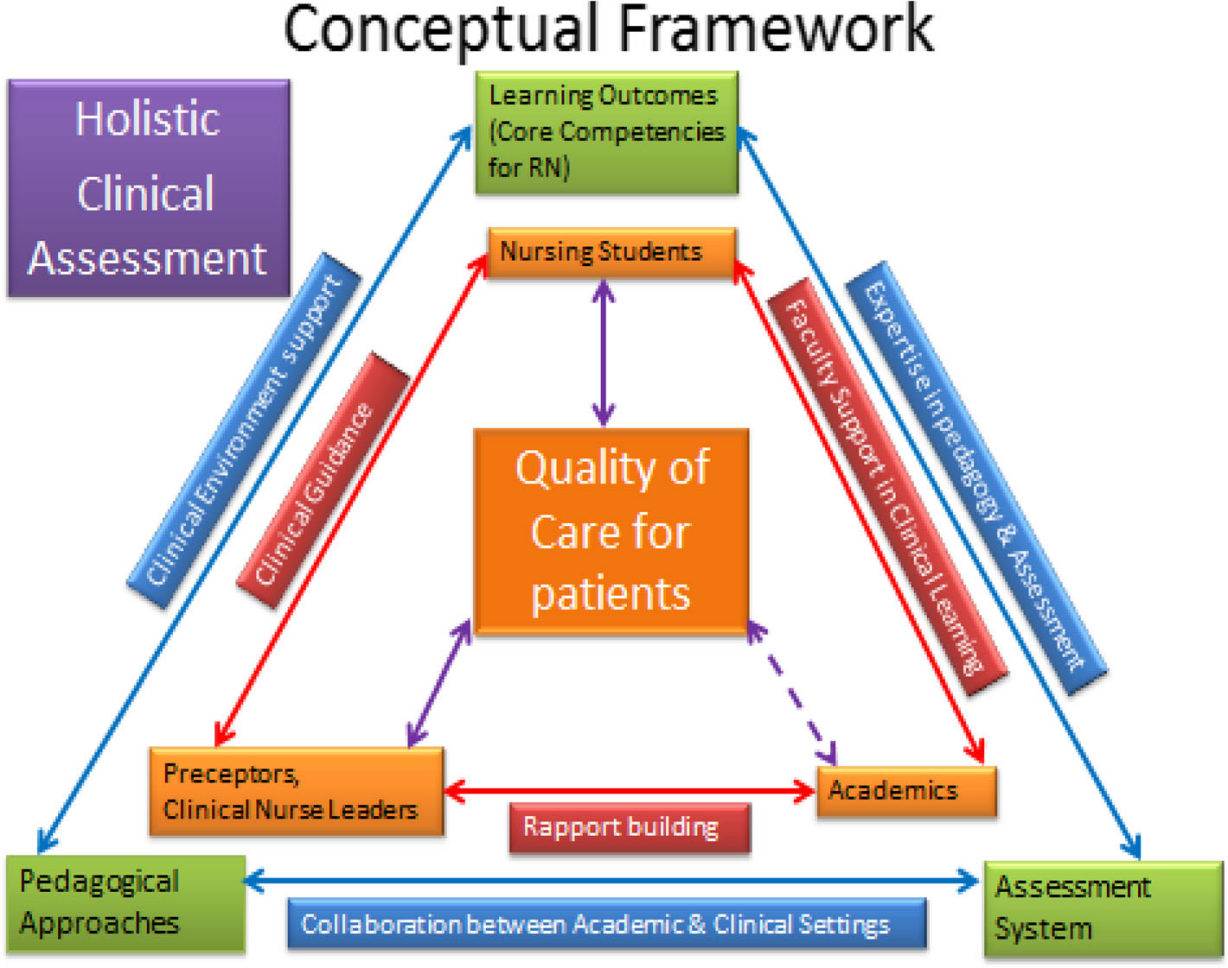
Conceptual Framework-Holistic Clinical Assessment [16, 18, 19]

The study’s conceptual framework was formed by making a theoretical connection of learning, pedagogy and assessment as advocated by Shepard’s [17] emergent paradigm (Fig. 1). A holistic assessment model promotes assessment for learning, rather than assessment of learning. However, change in assessment is never a stand-alone initiative; it has to be integrated with the curriculum, pedagogy and learning [17]. Experiential learning requires focused attentiveness on the part of the learner, a recognition that the practice itself is a continuous source of knowledge development and skill acquisition, and an environment where reflecting on the experience is deliberately planned [18]. Therefore, students’ learning, pedagogical approaches and assessment systems could be integrated to promote holistic learning experiences.

Preceptors invest long periods of time with students in the clinical setting. Clinical immersion happens rapidly as the student moves from shadowing the preceptor to performing nursing activities [19]. In fact, students and preceptors often develop an interactive and collaborative relationship. When nursing academics visit the hospital, they can assess the student/preceptor relationship, provide support to preceptors in terms of pedagogical approaches, motivate and encourage students to maximise their learning opportunities, and evaluate students’ clinical competence. The regular visits promote dialogue among students, preceptors and academics, and provide opportunities to discuss clinical teaching pedagogy, concerns in clinical practice, and areas for professional growth [20].

This study aimed to develop a Holistic Clinical Assessment Tool (HCAT) and test the psychometric properties of this newly developed tool. The study was conducted in a university and two tertiary health care settings in Singapore.

## Methods

### Development of holistic clinical assessment tool

A systematic review was conducted to explore the clinical assessment tools for nursing students [2]. The results indicated that most assessment tools are criterion-referenced using the standards from the respective National Board of Nursing from the various countries [2]. However, a limited number of studies have adequately tested the psychometric properties of the tools. As pointed out in the systematic review, clinical assessment requires collaboration among students, preceptors, clinical administrators and academics. Therefore, understanding the perspective of each key player is determinant factor for developing a holistic clinical assessment system. An explorative qualitative approach using focus group discussion was adopted. Ten focus groups discussions were conducted at various hospitals and university. The focus groups generated rich and deep discussions, and this 360° approach reflected the key players’ perspective on clinical assessment. The themes and subthemes that emerged from the focus group discussion were reported elsewhere and were considered significantly in the development of the HCAT [12, 21].

The core competencies of a RN, as defined by the Singapore Nursing Board [11], Nursing and Midwifery Council of United Kingdom [22], American Association of College of Nursing [7] and Nursing and Midwifery Board of Australia [10], were critically examined and reviewed. The assessment items and behavioural cues were developed with reference to the core competencies and in consideration of local situations [23]. The results of the qualitative studies identified indicators to strengthen the assessment framework and enhance support to students and preceptors. Based on these indicators and core competencies, critical contents were constructed as core components of the HCAT.

### Assessment of face and content validity

The initial version of HCAT consists of 4 domains, 39 items, one global rating scale, and behavioural cues to provide specific performance indicators. The items reflected knowledge, skills and attitudes required of undergraduate nursing students in the clinical context. The advisory committee were invited to critically evaluate whether HCAT would be able to assess nursing students’ clinical competencies holistically, and examine the format of the HCAT regarding clarity and ease of use. The advisory committee consisted of two nursing academics, one medical education academic, one academic specialised in psychometric testing, two hospital nurse administrators, one assessment specialist, and one education specialist. After multiple consultation rounds, consensus was achieved. The face validity of HCAT was established through consultation with key academic and clinical stakeholders.

A panel of content experts consisting of 4 preceptors, 2 nurse managers, 2 nurse educators, 3 academics and 3 nursing graduates was then invited to evaluate the HCAT. The panel members were invited to rate the content relevancy, clarity, conciseness, ambiguity and appropriateness of each assessment item using a 4-point rating scale: 1 = not relevant, 2 = somewhat relevant, 3 = relevant, and 4 = very relevant. Content Validity Index (CVI) is a plausible method of estimating the content validity of a new scale [24]. The Item Content Validity Index (I-CVI) and Scale Content Validity Index (S-CVI) were calculated. In the first round of content validity testing, two items were eliminated due to low I-CVI <0.65. One item was separated into two items as it consisted of multiple constructs, and 6 items were revised. The revised tool was sent to the experts for a second round of evaluation, which resulted in I-CVI = 1.00 for all the revised items and the final S-CVI = 0.9793.

### Pilot testing

A pilot test of the HCAT was then conducted with 20 final-year undergraduate nursing students at a tertiary university in Singapore. Students assessed their own clinical competencies using the HCAT. In addition, they provided feedback on the clarity of HCAT. The results of pilot test indicated that the internal consistency was satisfactory for the overall HCAT (α = .965), and 4 subscales: professional, legal and ethical nursing practice (α = .886), management of care (α = .938), leadership and nursing management (α = .851), and professional development (α = .844). No revision on the items was required based on the results of the pilot testing. The HCAT consist of 38 items grouped into 4 subscales.

### Psychometric testing

#### Data collection procedure

Psychometric testing was conducted to examine the newly developed HCAT, including the factor structure, internal consistency reliability and test-retest reliability. A population-based sample including a total of 130 final-year nursing students who have completed their Transition to Practice clinical attachment was recruited. The students were invited to self-evaluate their clinical competence using the HCAT. Demographic data (e.g. age, gender and ethnicity) was also obtained. To investigate test-retest reliability, 30 students from the primary group were invited to complete the HCAT two weeks after the first evaluation.

#### Data analysis

Data were analysed using SPSS Version 22.0. Data were examined for missing values. Six cases had missing data spread across 13 items. Missing data was imputed with the mean response for the respective item. An exploratory factor analysis (EFA) was conducted to examine the underlying dimensions of the HCAT. EFA is a powerful and elegant statistical technique to address an essential scientific goal of elucidating the underlying meaning of concepts of a newly developed tool [16]. Internal consistency was evaluated using Cronbach’s alpha and item-to-total correlation. Cronbach’s alpha coefficient indicates how well a group of items together measure the trait of interest [16]. The item-to-total correlation, which assesses the extent to which an item is related to the remainder of its subscale with the item omitted, should exceed .40 [25]. Test-retest reliability was evaluated using intraclass correlation coefficient (ICC). The scores from different time points can then be correlated to evaluate the stability of the tested tool over time [16].

## Results

### Characteristics of participants

A total of 130 final-year nursing students participated in the study. The nursing students were aged 21–24 years (mean = 22.83). Eighty-three percent (*n* = 108) were female. The students’ clinical practice settings varied widely in the major tertiary hospitals in Singapore, including Medical/Surgical wards (42 %, *n* = 55); Cardiology/Respiratory wards (11 %, *n* = 14); Oncology and Palliative wards (12 %, *n* = 12); Geriatric wards (8 %, *n* = 10); Orthopedic wards (6 %, *n* = 8); Paediatric wards (6 %, *n* = 8); Obstetrics/Gynaecology wards (6 %, *n* = 8); Emergency Departments (6 %, *n* = 8); and Neurology wards (5 %, *n* = 7).

### Factor structure

The Kaiser-Meyer-Olkin (KMO) coefficient was .897, and Bartlett’s test results reached statistical significance (x^2^(630) = 3195.57; *p* < 0.001), indicating the data being appropriate for conducting an EFA [16]. Based on the four domains of the Core Competencies for Registered Nurses [11], four fixed-factor solution using principal components analysis was examined. The four-factor solution explained 43.7, 6.7, 4.2, and 4.0 % of the variance respectively. The item loading was then tested using the rotated component matrix.

The four factors were interpretable using a loading criterion of .4 as a cut-off point [16]. All 38 items exhibited loadings >.4. Item 20 cross-loaded on three factors, and Item 19 crossed-loaded on two factors. Therefore, the two items were eliminated. Item 13 was loaded highest on Factor 1 and Item 21 loaded highest on Factor 3. Item 5, 7, 17, and 25 crossed-loaded on two factors. However, Item 5 and 7 carried a heavy loading on Factor 1; Item 17 loaded significantly higher on Factor 2; and Item 25 loaded higher on Factor 3. Therefore, the four items were retained in the original domains.

**Fig. 2:**
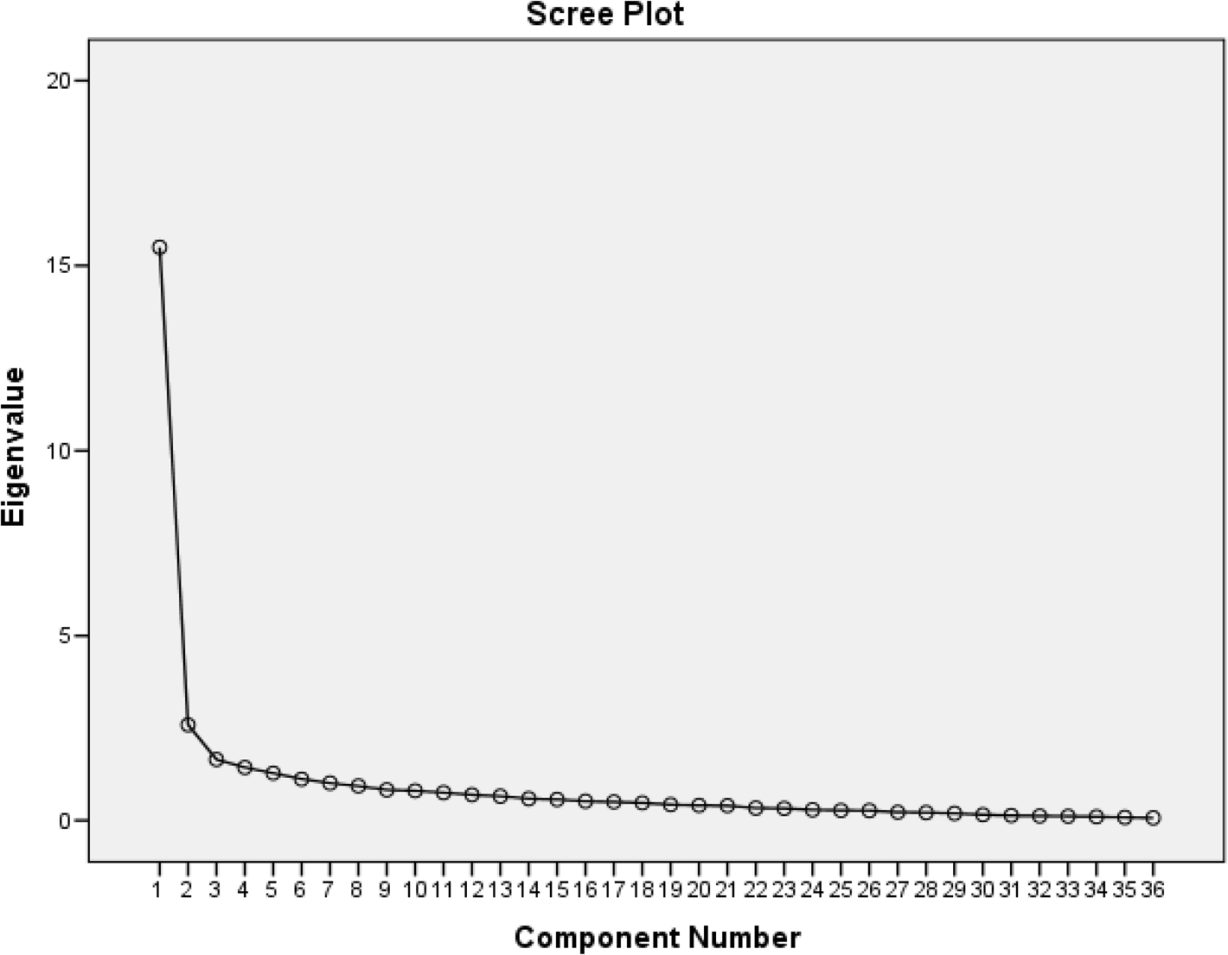
Screen plot of the factor loading

At the final stage, a priniciple components analysis (PCA) of the remaining 36 items was conducted, with four factors explaining 58.8 % of the total variance. The four factors explained 43.1, 7.2, 4.6 and 4.0 % of the variance respectively. The screen plot of the factor loading is presented in Fig. 2. Items had a loading of >.4 or higher for each component are indicated in Table 1. The items are ordered and grouped by factors to facilitate interpretation. The four-factor model generally reflected the dimensions underlying the subscales, which are the domains identified as core competencies.

**Table 1:** Rotated Component Matrix^a^ of four factors for HCAT (factor loadings >.40)

Domains and assessment items	Component			
	1	2	3	4
1. Professional, legal and ethical nursing practice				
1) Complies with Singapore Nursing Board (SNB) Code of Ethics and Professional Conduct, Standards of Practice for nurses and midwives	.614	.322	.195	.191
2) Practises with reference to institutional/national legislation, policies and procedural guidelines	.570	.338	.317	.201
3) Demonstrates responsibility and accountability for care within scope of practice and level of competence	.632	.246	.330	.250
4) Complies with professional expectations	.608	.242	.364	.307
5) Works with Registered Nurse (RN) to apply SNB Code of Ethics and Professional Conduct for ethical decision making	.568	.340	.171	.415
6) Works with RN to discuss care provision with the client and family within a reasonable time frame	.495	.359	.116	.315
7) Seeks permission from the client in the delivery of care	.617	.166	.175	.389
8) Respects the values of individuals	.722	.126	.285	.229
9) Respects the cultural practices of individuals	.819	.047	.122	.106
10) Respects the individual’s religious beliefs and practices, as well as their spirituality needs	.850	.101	.095	.079
13) Shows caring attributes towards clients and families	.519	.358	.133	.039
2. Management of care				
11) Communicates effectively and timely using appropriate verbal skills with clients and families	.311	.418	.231	.122
12) Communicates effectively using appropriate non-verbal skills with clients and families	.388	.487	.305	.069
14) Performs comprehensive and systematic assessment	.192	.694	.312	.186
15) Formulates plans of care with the healthcare team, client and families	.358	.661	.199	.114
16) Implements the holistic plan of care safely and timely	.157	.716	.357	.177
17) Evaluates and modifies plan of care with appropriate documentation	.091	.636	.263	.397
18) Applies critical thinking skills and makes appropriate clinical decisions	.136	.671	.337	.145
22) Conducts educational needs assessment	.155	.754	.299	.180
23) Empowers aspects of care to clients, families and carers	.165	.674	.190	.183
24) Provides information using available resources	.299	.688	.285	.086
3. Leadership & nursing management				
25) Demonstrates collaborative practice with healthcare professionals	.448	.287	.493	−.040
26) Establishes rapport and interacts with team members in a supportive manner	.394	.120	.586	.033
27) Demonstrates effective delegation to team members	.170	.312	.560	−.001
28) Follows up on the work delegated	.292	.278	.612	.197
29) Utilises materials efficiently and minimises wastage	.102	.265	.592	.300
30) Works with RN to make efficient use of manpower resources	.023	.346	.649	.328
31) Manages time in an effective manner	.047	.350	.739	.040
21) Provides a safe care environment for clients	.325	.192	.490	.368
32) Demonstrates knowledge of occupational health and safety policies and procedures	.217	.371	.566	.140
33) Prioritises the tasks based on the urgency of the clinical situation	.241	.355	.646	.055
34) Manages workloads effectively by seeking help when necessary	.252	.088	.666	.174
4. Professional development				
35) Reflects on own nursing practice	.284	.371	−.029	.555
36) Responds positively to constructive feedback	.255	.189	.148	.769
37) Takes steps to address areas of improvement in skills and knowledge	.187	.111	.187	.780
38) Demonstrates basic knowledge on evidence-based practice	.278	.221	.390	.495

Extraction Method: Principal Component Analysis. Rotation Method: Varimax with Kaiser Normalisation^a^

a. Rotation converged in seven iterations

### Reliability

The Cronbach’s alpha for the total scale was .965, and each subscale of HCAT were .924, .916, .909 and .789 respectively. All 36 items showed satisfactory item-to-total correlation, ranging from .519 to .781 (Additional file 1: Table S2). Thirty students completed the HCAT two weeks after the first evaluation. The test-retest reliability was satisfactory with ICC of .979 for the total scale, and ICC scores of .957, .938, .927, and .881 for the four subscales (Additional file 1: Table S2).

## Discussion

This study focused on the development and psychometric testing of the HCAT. The systematic review, qualitative studies and comparisons of core competencies generated a comprehensive pool of items. The sample of participants provided the opportunity for testing the psychometric properties of the instrument. The results indicated that HCAT has a four-factor structure, and satisfied level of internal consistency. In addition, the test-retest reliability reconfirmed the stability of the overall scale and four subscales. This rigorous process of tool development and psychometric testing ensures the validity and reliability of the HCAT.

The four-factor structure of the 36-item HCAT was robust and the domains reflect clinical competence required for nursing undergraduates. In the realm of Professional, Legal and Ethical Nursing Practice, the ten items were all loaded in their original factor. It appears that all these items reflected the expected behaviour of a professional nurse. Interestingly, Item 13, “shows caring attributes towards clients and families”, which was formerly incorporated in the Management of Care subscale, demonstrated the highest loading on the Professional, Legal and Ethical Nursing Practice subscale (.519). This result suggests that caring attributes is also a crucial aspect of professional nursing practice. Indeed, the caring attributes of a nurse has been discussed in the scholarly work of Carper [26] as “the art of nursing”.

The ten items emerged from the Management of Care subscale, and these items typify the knowledge, clinical, communication and critical thinking skills built on clinical exposure and experience [27]. Hassmiller [28] recommends that nurses must develop basic competencies to meet the demands of dynamic clinical situations, with a focus on clinical preparation, emphasising quality and safety, evidence-based practice and leadership. Item 19 and 20, “identifies potential clinical risks through clinical risk assessment tools”, and “identifies risk management strategies to maintain safe care environment”, were cross-loaded on two to three factors. These items were removed from this subscale. However, these items still have face validity, and relevant contents were added to the behavioural cues to illustrate the nursing management of a safe environment. It is interesting to note that certain items were loaded in two factors (Item 5, 7, 17, 25), which reconciles with the fact that nursing practice is complex and requires the combination of knowledge, psychomotor skills, attitudes and critical thinking skills [29]. Allocating the items to a factor according to the higher item loading coefficient could be a reasonable approach [30].

In this study, the ten items included in the Leadership and Nursing Management subscale focus on managerial and leadership skills, resource utilisation, and a safe working environment. Item 21, “provides a safe care environment for clients”, and was previously included in the subscale of Management of Care. In fact, this item demonstrated the highest loading of .490 on Factor 3. The empirical results suggest that nursing leadership encompasses management of people, resources and environment. It has been acknowledged in the literature that leadership qualities of nurses impact the possibility of sustainable, safe, and high-quality care. Furthermore, highly skilled practice and increasing dependence on the nurse’s role in coordination and management promotes the continuum of care [31].

The four items loaded within the domain of Professional Development were original. This factor focuses on the behavioural characteristics of continuous self-development in the nursing profession. In particular, the items describe reflection on nursing practice, responding positively to feedback and application of evidence-based practice. The relationship between the individual items and the factor was robust as the individual item loadings ranged from .495 to .780. These results imply that nursing undergraduates believed that continuous professional development was an essential component in clinical competence. Notably, it is increasingly brought to the awareness that nurses need to engage in evidence-based practice to provide holistic care to patients [32]. Scholars have been working on the development of clinical competence in various nursing contexts. Despite contextual variation, it is acknowledged that the development of clinical competence is complex and it is imperative to assess competence in a more holistic manner [29].

The PCA results were confirmed by computing reliability estimates. The Cronbach’s alpha estimates for the four subscales and the total scale were satisfactory. In addition, the results of ICC were acceptable. Thus, the test-retest method reconfirmed the stability of the HCAT. These empirical results added further credence to the robustness of the PCA results.

The ultimate goal of clinical education is the development of nursing students who are confident and competent beginning practitioners. HCAT captures the essential domains of clinical competence in nursing students, and focuses on holistic learning and assessment in the clinical environment. The results of the study have indicated that the HCAT is a reliable and valid clinical assessment tool for evaluating nursing students’ clinical competency holistically. Furthermore, the use of HCAT facilitates self-reflection and monitoring of one’s clinical progress. In addition, the HCAT can be used for peer-assessment and feedback. Jackson and Larkin [33] opined that peer-assessment is necessary to help students reflect on their performance; through such reflections, students are able to locate their strengths and weaknesses, determine a better way to approach a task, and learn the necessary information to perform a task. It is noteworthy that the HCAT can be used as a guide for preceptors in the process of clinical assessment and providing constructive feedback. The behavioural cues provide a useful guide in gauging how well a student is coping and how one can improve holistically. However, the 36-item HCAT may bring additional workload to the preceptors. Stress and workload of preceptors are one of the increasing concerns about the quality of clinical experience in other countries as well [34]. Nevertheless, Cowan et al. [30] emphasised that a balance is needed between the too-lengthy competency assessment or too-narrow assessment which would not be sufficiently comprehensive.

The systematic review indicated that the clinical assessment processes and tools varied across different countries [2]. Cassidy et al. [35] have found that the competency assessment in Ireland consists of preliminary, intermediate, and final interviews between the student and the preceptor. Löfmark and Thorell-Ekstrand [36] have found that, in Sweden, the students and preceptors have discussions at the midpoint and the end of the clinical period, to provide continuous feedback with the Assessment Form for Clinical Nursing Education. In Australia, the Structured Observation and Assessment of Practice includes observing the student’s engagement with patient-care activities, and the provision of formative and summative feedback by the clinical educators to the students [37]. O’Connor et al. [38] have investigated the need for students and preceptors to follow a protocol-Shared Specialist Placement Document, a generic assessment tool that encompasses standards of practice and indicators of competence in Ireland.

Although varies assessment processes and tools are practised in different countries, it is important to consider the local situation carefully. It is essential to discuss with the clinical partners and explore the most appropriate process to perform the clinical assessment. Since the HCAT is psychometrically demonstrated to be valid and reliable, its utility may be considered in other institutions. Modification to the HCAT could be done based on the local context, followed by validation through psychometric testing of the revised assessment tool for the different context. The goal is to facilitate the learning of the students in clinical settings and to assist them to achieve the required level of clinical competence.

### Recommendations for future research

As instrument development is an iterative process, further studies should be conducted on a larger cohort at various locations. Besides using HCAT for self-assessment and peer-assessment, preceptors could use HCAT to evaluate students’ clinical competency. Thus, future studies could explore the inter-rater reliability of the HCAT when used by preceptors to examine the relationship between perceived and actual competence, which would further strengthen the convergent validity of the HCAT. In addition, confirmatory factor analysis could be conducted with a greater sample size to further validate the construct of the HCAT.

### Limitations of the study

It is acknowledged that the relatively small sample size and use of convenience sampling in this study may limit the generalisability of the findings. In addition, clinical competence was measured through the perception of nursing students rather than the actual demonstration of competent behaviours. Despite researchers’ concerns about the outcomes of using self-reported measures of competence, self-assessment could be used for reflective practice, and support other ways of assessment [39]. Furthermore, the Cronbach alpha of .965 for the total scale may suggest that different factors are not so distinct.

## Conclusions

This study has contributed to advancing the development and measurement of a tool for assessing clinical competence. The results of this study indicate that HCAT is both reliable and valid. HCAT has the potential to be used as a valid measure to evaluate clinical competence in nursing students, and provide specific and ongoing feedback to enhance the holistic clinical learning experience. In addition, HCAT functions as a tool for self-reflection, peer-assessment, and guides preceptors in clinical teaching and assessment. Above all, this study supports HCAT as a tool that delineates the knowledge, skills and professional attributes of each competency domain that are crucial for students to develop and transfer from theory to practice to ensure they provide safe and quality care upon graduation.

## Additional file

Additional file 1: Table S2. Internal consistency (Item-to-total correlations and Cronbach’s α) and Test-retest reliability of the HCAT. (PDF 232 kb)

## Abbreviations

CVI: Content validity index
EFA: Exploratory factor analysis
HCAT: Holistic clinical assessment tool
ICC: Intraclass correlation coefficient
I-CVI: Item content validity index
KMO: Kaiser-Meyer-Olkin coefficient
PCA: Priniciple components analysis
RN: Registered nurse
S-CVI: Scale content validity index
