# Supplementary material for: Development and psychometric testing of Holistic Clinical Assessment Tool (HCAT) for undergraduate nursing students
Source: BMC Med Educ. 2016 Sep 22;16:248. doi: 10.1186/s12909-016-0768-0 (PMC5034523; doi:10.1186/s12909-016-0768-0)
Supplement: Additional file 1: Table S2. — Internal consistency (Item-to-total correlations and Cronbach’s α) and Test-retest reliability of the HCAT. (PDF 232 kb) [file 12909_2016_768_MOESM1_ESM.pdf]

Table 2: Internal consistency (Item-to-total correlations and Cronbach's α) and Test-retest reliability of the HCAT

Domains and Assessment items	Item-Total Correlation (n = 130)	Cronbach's Alpha (n = 130)	Intra-class Correlation Coefficient (n = 30)
1.Professional, legal and ethical nursing practice		.924	.957
1) Complies with Singapore Nursing Board (SNB) Code of Ethics and Professional Conduct, Standards of Practice for nurses and midwives	.716		
2) Practises with reference to institutional/national legislation, policies and procedural guidelines	.697		
3) Demonstrates responsibility and accountability for care within scope of practice and level of competence	.750		
4) Complies with professional expectations	.743		
5) Works with Registered Nurse (RN) to apply SNB Code of Ethics and Professional Conduct for ethical decision making	.705		
6) Works with RN to discuss care provision with the client and family within a reasonable time frame	.619		
7) Seeks permission from the client in the delivery of care	.713		
8) Respects the values of individuals	.754		
9) Respects the cultural practices of individuals	.681		
10) Respects the individual's religious beliefs and practices, as well as their spirituality needs	.706		
13) Shows caring attributes towards clients and families	.558		
2.Management of care		.916	.938
11) Communicates effectively and timely using appropriate verbal skills with clients and families	.524		
12) Communicates effectively using appropriate non-verbal skills with clients and families	.588		
14) Performs comprehensive and systematic assessment	.761		
15) Formulates plans of care with the healthcare team, client and families	.676		
16) Implements the holistic plan of care safely and timely	.775		
17) Evaluates and modifies plan of care with appropriate documentation	.704		
18) Applies critical thinking skills and makes appropriate clinical decisions	.708		
22) Conducts educational needs assessment	.781		
23) Empowers aspects of care to clients, families and carers	.645		
24) Provides information using available resources	.731		

3. Leadership & nursing management		.909	.927
25) Demonstrates collaborative practice with healthcare professionals	.594		
26) Establishes rapport and interacts with team members in a supportive manner	.621		
27) Demonstrates effective delegation to team members	.563		
28) Follows up on the work delegated	.702		
29) Utilises materials efficiently and minimises wastage	.641		
30) Works with RN to make efficient use of manpower resources	.709		
31) Manages time in an effective manner	.725		
21) Provides a safe care environment for clients	.618		
32) Demonstrates knowledge of occupational health and safety policies and procedures	.662		
33) Prioritises the tasks based on the urgency of the clinical situation	.723		
34) Manages workloads effectively by seeking help when necessary	.652		
4. Professional development		.789	.881
35) Reflects on own nursing practice	.543		
36) Responds positively to constructive feedback	.664		
37) Takes steps to address areas of improvement in skills and knowledge	.673		
38) Demonstrates basic knowledge on evidence-based practice	.519		
**Total score**		.965	.979
